# *Lacticaseibacillus rhamnosus* CRL1505 Ameliorates Liver Injury and Inflammation in Poly(I:C)-Induced Acute Hepatitis

**DOI:** 10.3390/foods15061034

**Published:** 2026-03-16

**Authors:** María José Lorenzo Pisarello, Ayelen Antonella Baillo, Mariano Elean, Leonardo Albarracín, Luciano Arellano-Arriagada, Yoshihito Suda, Haruki Kitazawa, Julio Villena

**Affiliations:** 1 Laboratory of Immunobiotechnology, Reference Centre for Lactobacilli (CERELA-CONICET), San Miguel de Tucuman 4000, Tucuman, Argentina; mjlorenzopisarello@cerela.org.ar (M.J.L.P.); abaillo@cerela.org.ar (A.A.B.); melean@cerela.org.ar (M.E.); 2 Laboratory of Respiratory Immunology (LaRI), Division of Animal Immunology and Omics, International Education and Research Center for Food and Agricultural Immunology (CFAI), Graduate School of Agricultural Science, Tohoku University, Sendai 980-8572, Japan; lalbarracin@herrera.unt.edu.ar (L.A.); luciano.andres.arellano.arriagada.r1@dc.tohoku.ac.jp (L.A.-A.); 3 Food and Feed Immunology Group, Laboratory of Animal Food Function, Graduate School of Agricultural Science, Tohoku University, Sendai 980-8572, Japan; 4 Department of Food, Agriculture and Environment, Miyagi University, Sendai 980-8572, Japan; suda@myu.ac.jp; 5 Livestock Immunology Unit, International Education and Research Centre for Food and Agricultural Immunology (CFAI), Graduate School of Agricultural Science, Tohoku University, Sendai 980-8572, Japan

**Keywords:** liver injury, immunobiotic, *Lacticaseibacillus rhamnosus* CRL1505, TLR3, hepatitis

## Abstract

*Lacticaseibacillus rhamnosus* CRL1505 enhances antiviral immunity at mucosal sites, but its capacity to modulate liver immune responses remains unclear. Therefore, this study evaluated whether this immunomodulatory bacterium protects against Toll-like receptor 3 (TLR3)-mediated acute hepatitis induced by poly(I:C), and whether this effect depends on mucosal adhesion. BALB/c mice received the wild-type CRL1505 strain or the Δ*mbf* CRL1505 mutant lacking the mucus-binding factor gene prior to poly(I:C) challenge. Liver injury, serum transaminases, and hepatic expression of interferons (IFNs), antiviral factors, inflammatory mediators, and regulatory cytokines were evaluated 48 h later. Poly(I:C) challenge induced acute hepatitis characterized by increased ALT/AST levels, leukocyte infiltration, and elevated hepatic IFNs and proinflammatory cytokines. The CRL1505 strain administration significantly reduced TNF-α, IL-1β, and IL-6 while enhancing IFNs, antiviral factors, and the regulatory cytokines IL-10 and IL-27, resulting in improved transaminase levels and attenuated liver damage. Notably, the Δ*mbf* CRL1505 mutant conferred protection comparable to the wild-type strain. These findings demonstrate that *L. rhamnosus* CRL1505 exerts immunomodulatory and hepatoprotective effects during TLR3-driven hepatitis and that mbf-mediated adhesion is not required for this protection. Overall, CRL1505 emerges as a promising preventive strategy to enhance antiviral defenses and limit inflammation-associated liver injury.

## 1. Introduction

Liver injury can arise from a wide variety of causes, including viral infections, pharmacological agents, and excessive alcohol consumption. These insults initiate a spectrum of liver disorders that often begin with acute liver injury [1], which is a clinically significant syndrome characterized by jaundice, coagulopathy, and hepatic encephalopathy, that can rapidly progress to acute liver failure within six months. Acute liver failure is associated with multi-organ complications and high mortality rates [1]. Moreover, patients who survive acute liver failure remain at risk of developing chronic liver diseases such as cirrhosis and hepatocellular carcinoma, both of which pose major public health challenges worldwide [2].

Inflammation is a central driver of liver disease pathogenesis and plays a decisive role in its progression [3,4]. Immune-mediated hepatitis arises from complex interactions between hepatic parenchymal and non-parenchymal cells and multiple immune cell populations in response to injurious stimuli, including viral infections. These interactions disrupt hepatic homeostasis and initiate a cascade of inflammatory events characterized by the production of cytokines and chemokines, such as tumor necrosis factor-α (TNF-α) and interferon-γ (IFN-γ) [5,6]. Among the innate immune mechanisms that link viral sensing to hepatic inflammation, Toll-like receptor 3 (TLR3) plays a pivotal role. Viruses are a major cause of liver disease, with hepatitis A, B, C, D, and E viruses (HAV, HBV, HCV, HDV, and HEV, respectively) representing the principal hepatotropic pathogens [5,7]. The diversity in their clinical courses and outcomes reflects both virus-specific features and the nature of the host immune responses they elicit [5,7]. In addition to the cytopathic effect inherent to viral replication, viral components such as proteins and nucleic acids can also cause damage to the host tissue through the generation of dysregulated inflammatory responses. In this sense, it was shown that viral double-stranded RNA (dsRNA), or synthetic dsRNA analogs such as polyinosinic:polycytidylic acid [poly(I:C)], which are recognized by TLR3 expressed in hepatic and immune cells, can drive hepatic inflammation [5,6].

Upon dsRNA engagement, TLR3 signals exclusively through the TIR domain-containing adaptor inducing interferon-β (TRIF), thereby initiating distinct downstream signaling pathways. The TRIF-dependent cascade involves the recruitment of tumor necrosis factor receptor-associated factors 3 and 6 (TRAF3/6), TANK-binding kinase 1 (TBK1), IκB kinase ε (IKKε/IKK-i), receptor-interacting protein 1 (RIP1), and nuclear factor-κB (NF-κB)-activating kinase-associated protein 1 (NAP1). These signaling modules converge on the activation and nuclear translocation of key transcription factors, including interferon regulatory factors 3 and 7 (IRF3/7), NF-κB, and activator protein 1 (AP-1) [7,8,9]. Activation of these transcriptional programs drives the coordinated expression of type I IFNs, proinflammatory cytokines, and chemokines, which are essential for antiviral defense but may also contribute to immune-mediated liver injury [7,8,9]. Thus, TLR3-dependent signaling constitutes a critical molecular link between viral recognition and inflammation-associated hepatic damage, providing a strong mechanistic rationale for experimental models of viral hepatitis based on synthetic dsRNA stimulation, as originally demonstrated in TLR3-deficient mice and subsequently validated in experimental models of acute hepatitis [10,11,12].

Given the central role of inflammation in hepatic diseases, increasing attention has been directed toward the gut–liver axis, a bidirectional communication network linking intestinal and hepatic immunity that contributes substantially to the onset and progression of many liver disorders [13,14,15,16,17,18,19]. Within this framework, probiotics with immunomodulatory properties, termed immunobiotics, have emerged as promising candidates for restoring immune homeostasis and reducing liver injury [1,17,18,20]. Several species of the lactobacilli group have been shown to alleviate hepatic inflammation and tissue damage in diverse models, including alcoholic liver disease and acute liver injury induced by D-galactosamine or acetaminophen [21,22,23,24,25,26,27,28]. Numerous studies have demonstrated that probiotics can modulate hepatic inflammation through pattern recognition receptors such as TLR4, TLR2, TLR5, and TLR9, particularly in experimental models of alcohol-induced liver injury, carbon tetrachloride (CCl_4_) toxicity, non-alcoholic fatty liver disease (NAFLD), and autoimmune hepatitis [23,29,30,31,32,33,34]. However, none of these studies have specifically examined the involvement of the hepatic TLR3 signaling pathway. Although substantial evidence indicates that certain probiotic strains are capable of regulating TLR3-dependent immune responses in the intestine [35,36,37], thereby supporting the relevance of this pathway as a working hypothesis, its role as a mechanistic target in the liver has not been previously established. The present study therefore provides the first direct evidence that an immunobiotic strain modulates hepatic inflammation specifically triggered by TLR3 activation.

*Lacticaseibacillus rhamnosus* CRL1505 is a particularly well-characterized immunobiotic capable of modulating both innate and adaptive immune responses in the gut and systemically [38]. Experimental and clinical studies demonstrate that *L. rhamnosus* CRL1505 regulates cytokine production, enhances the activity of innate immune cells, and helps shape controlled inflammatory responses that improve host resistance to infectious and inflammatory insults [39,40,41,42]. Notably, previous research has shown that orally administered *L. rhamnosus* CRL1505 enhances TLR3-mediated antiviral responses in intestinal epithelial cells, promoting the expression of type I IFNs (IFN-β), IFN-γ and other antiviral mediators [36]. Although most evidence characterizing *L. rhamnosus* CRL1505’s effects derives from mucosal tissues, its ability to modulate systemic immune responses suggests potential benefits in extraintestinal organs such as the liver. However, the potential beneficial role of the CRL1505 strain in the TLR3-mediated inflammation in the liver has not been investigated before.

In this study, we evaluated whether the oral administration of *L. rhamnosus* CRL1505 to mice can ameliorate poly(I:C)-induced acute hepatitis and associated inflammatory responses. In addition, we studied the immunomodulatory potential of *L. rhamnosus* Δ*mbf* CRL1505—a knockout mutant lacking the mbf gene involved in intestinal mucosal adhesion [38]—to determine whether the ability to modulate liver inflammation through the gut-liver axis depends on this adhesion-associated factor.

## 2. Materials and Methods

### 2.1. Microorganisms

*Lacticaseibacillus rhamnosus* CRL1505 was obtained from the Reference Centre for Lactobacilli (CERELA-CONICET) culture collection (San Miguel de Tucuman, Tucuman, Argentina). A mutant of the CRL1505 strain lacking the expression of the mbf gene, referred to as *L. rhamnosus* Δ*mbf* CRL1505 was constructed as described previously [43]. Both wild-type and mutant strains were stored at −80 °C in MRS broth supplemented with 50% glycerol until use. For experiments, both strains were cultured under aerobic conditions in Man–Rogosa–Sharpe (MRS) broth (Oxoid, Cambridge, UK) at 37 °C for 12 h to reach the late logarithmic phase. Bacterial cells were harvested by centrifugation at 30,000× *g* for 10 min, washed three times with sterile 0.01 mol/L phosphate-buffered saline (PBS, pH 7.2), and finally resuspended in sterile 10% non-fat milk for oral administration to mice [37].

### 2.2. Animal Experimental Design

Twenty-four 6-week-old BALB/c mice were obtained from a closed colony maintained at CERELA-CONICET (Tucumán, Argentina) and were raised in a controlled atmosphere (22 ± 2 °C temperature, 55 ± 2% humidity) with a 12 h light/dark cycle. After acclimating for a week, all animals were randomly assigned to four experimental groups (*n* = 6 per group): (1) mock group, (2) control group, (3) wild-type *L. rhamnosus* CRL1505 group, and (4) *L. rhamnosus* Δ*mbf* CRL1505 group.

Immunobiotic CRL1505 or Δ*mbf* CRL1505 strains were resuspended in sterile 10% non-fat milk, prepared with sterile water, and administered to the respective groups for five consecutive days at a dose of 10^8^ cells/mouse/day in drinking water, following published data on the optimal immunoregulatory dose [44]. Control and mock groups received the same volume of sterile 10% non-fat milk without lactobacilli, ensuring identical vehicle exposure and handling conditions across all experimental groups.

After this administration period, CRL1505 and Δ*mbf* CRL1505 groups were injected intraperitoneally (IP) with 100 µL of PBS containing 30 µg of the TLR3 agonist poly(I:C). The control group also received an IP injection of poly(I:C), whereas the mock group received an equivalent volume of sterile PBS. All animals were maintained under identical housing conditions and received a conventional balanced diet and drinking water ad libitum. The innate antiviral immune response was evaluated 2 days after poly(I:C) stimulation. A schematic summary of the experimental design is provided in Supplementary Table S1.

In this study, the poly(I:C)-challenged group without immunobiotic treatment was considered the positive inflammatory control, as poly(I:C)-induced TLR3 activation is a well-established and reproducible model of acute inflammatory responses. The primary objective of the experimental design was to evaluate the preventive immunomodulatory capacity of CRL1505 in the context of TLR3 activation rather than to compare its effects with those of pharmacological anti-inflammatory agents or specific signaling inhibitors.

All experiments complied with the Guide for Care and Use of Laboratory Animals and were approved by the Ethical Committee of Animal Care at CERELA, Argentina (protocol number CRL-CICUAL-IBT-2024/4A).

### 2.3. Blood Sampling

All animals (*n* = 6 per group) were euthanized, and blood samples were collected through cardiac puncture at the end of each treatment and were collected in tubes containing EDTA as an anticoagulant. Samples were spun at 1500–2000 rpm for 15 min at room temperature to obtain serum. The resulting serum fractions were stored at −20 °C until subsequent biochemical and cytokine assessments.

### 2.4. Biochemical Analyses

Due to biochemical indication of liver function, levels of serum biomarkers alanine aminotransferase (ALT) and aspartate aminotransferase (AST) were estimated in all experimental groups (*n* = 6 per group) by a commercially available diagnostic kit of Wiener Lab (Rosario, Argentina). Enzyme activities were expressed as international units per liter (IU per L).

### 2.5. Hematoxylin and Eosin Staining

Liver samples from all experimental groups (*n* = 6 per group) were excised, washed with PBS, and immersed in 4% (*v*/*v*) formalin saline solution before routine paraffin embedding. The liver tissues were sectioned at a thickness of 5 μm and were stained with haematoxylin and eosin (H&E) to evaluate histopathological alterations associated with acute liver inflammation. Twenty areas per slice were examined, and representative images were presented. Pathological changes, including inflammatory cell infiltration, hepatocellular damage, and necrosis, were analyzed following Kleiner et al. [45]. Images were captured using an Axio Scope A1 fluorescence microscope (Carl Zeiss, Jena, Germany) equipped with a camera and processed with ZEN blue software (version 3.7.97.04000, Carl Zeiss Microscopy GmbH, Jena, Germany).

### 2.6. Quantification of Level of Serum Cytokines by ELISA

Serum TNF-α, IL-6, IL-10, and IFN-γ were measured in all experimental groups (*n* = 6 per group) using cytokines enzyme-linked immunosorbent assay (ELISA) technique assay kits (R&D Systems, Minneapolis, MN, USA), according to the manufacturer’s instructions.

### 2.7. Quantitative Expression Analysis by Real-Time PCR

The mRNA levels were determined in all experimental groups (*n* = 6 per group) using real-time PCR. Total RNA was isolated with TRIzol and reverse transcribed according to the manufacturer’s protocol. Two-step real-time quantitative PCR was performed to characterize the expression of *Tnf*, *Il6*, *Cxcl1*, *Il1b*, *Il10*, *Ifng*, *Ifnb1*, *Mx1*, *Oas1*, *Rnasel*, *Ifitm3*, *Ifnl2/3*, *Il27*, and *Ifnl1*. Expression of *Actb* (β actin) was used to normalize cDNA levels for differences in total cDNA levels in the samples. The primers were described previously [46]. The procedure was performed as described previously [46].

### 2.8. Statistical Analysis

Experiments were performed in triplicate and results were expressed as mean ± standard deviation (SD). After verification of the normal distribution of data, 2-way ANOVA was used. Tukey’s test (for pairwise comparisons of the means) was used to test for differences between the groups. Differences were considered significant at *p* < 0.05.

## 3. Results

### 3.1. Lacticaseibacillus rhamnosus CRL1505 Administration Ameliorates Liver Injury Induced by Poly(I:C)

**Figure 1:**
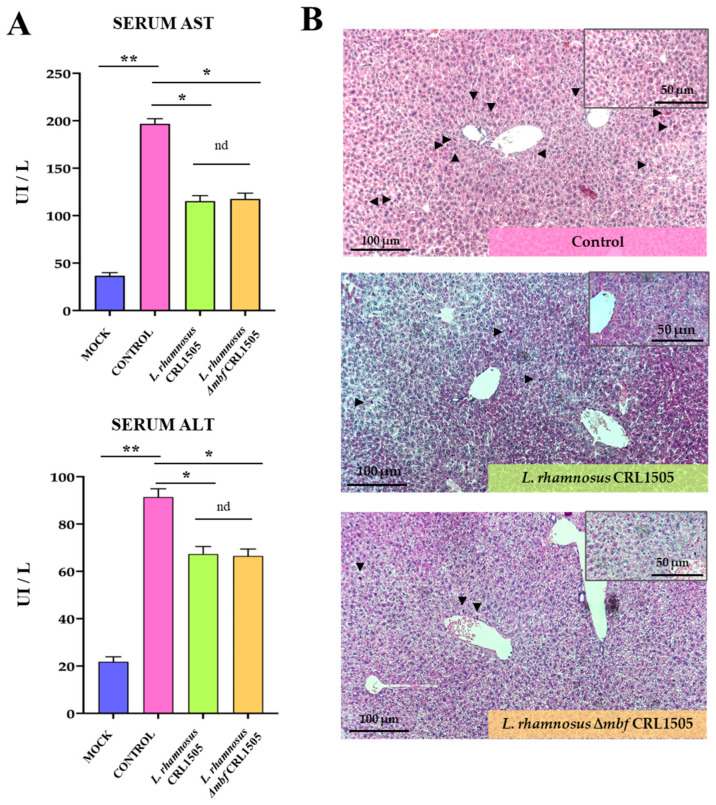
Effect of *Lacticaseibacillus rhamnosus* CRL1505 on liver injury induced by the activation of TLR3. BALB/c mice were orally treated with *L. rhamnosus* CRL1505 or Δ*mbf* strains for five days and then intraperitoneally challenged with poly(I:C). Mice without lactobacilli treatment and stimulated with poly(I:C) were used as controls. Forty-eight hours after TLR3 activation, (**A**) serum AST and ALT levels and (**B**) hematoxylin and eosin staining of liver sections were determined. Representative histological images are shown at 10× magnification; inset images are also shown at 10× magnification. Arrows indicate representative inflammatory cell infiltration. Data was presented as means ± SD of experimental groups and analyzed by ANOVA. Significant differences are shown compared to the control group at *p* < 0.05 (*), *p* < 0.01 (**), nd: no significant differences were detected.

To determine whether the poly(I:C)-induced liver injury could be reduced by the immunobiotic *L. rhamnosus* CRL1505, serum enzymes that are indicative of liver function were analyzed. The administration of poly(I:C) caused a significant elevation in the serum liver enzymes alanine aminotransferase (ALT) and aspartate aminotransferase (AST) (Figure 1A). Pretreatment with *L. rhamnosus* CRL1505 or *L. rhamnosus* Δ*mbf* CRL1505 significantly alleviated the hepatotoxic effect of poly(I:C) (Figure 1A). No significant differences were found between mice treated with the wild-type and mutant *L. rhamnosus* CRL1505 strains.

We also performed histological analysis of the liver tissue to identify any changes induced by the activation of TLR3 signaling pathway in the different experimental groups (Figure 1B). Based on the histopathological examination it was concluded that the intraperitoneal injection of poly(I:C) provoked moderate liver injury involving hepatic inflammation with cell infiltration, especially around the central veins in the control group (Figure 1B). As shown in the photomicrographs of hematoxylin and eosin staining, the treatment with both *L. rhamnosus* CRL1505 and *L. rhamnosus* Δ*mbf* CRL1505 alleviated liver inflammatory cells infiltration when compared to the control group. Oral administration of either bacteria, wild-type or mutant, showed the same effect on liver histology, with no differences found between the two groups (Figure 1B).

### 3.2. Lacticaseibacillus rhamnosus CRL1505 Administration Differentially Modulate Liver Cytokines Induced by Poly(I:C)

**Figure 2:**
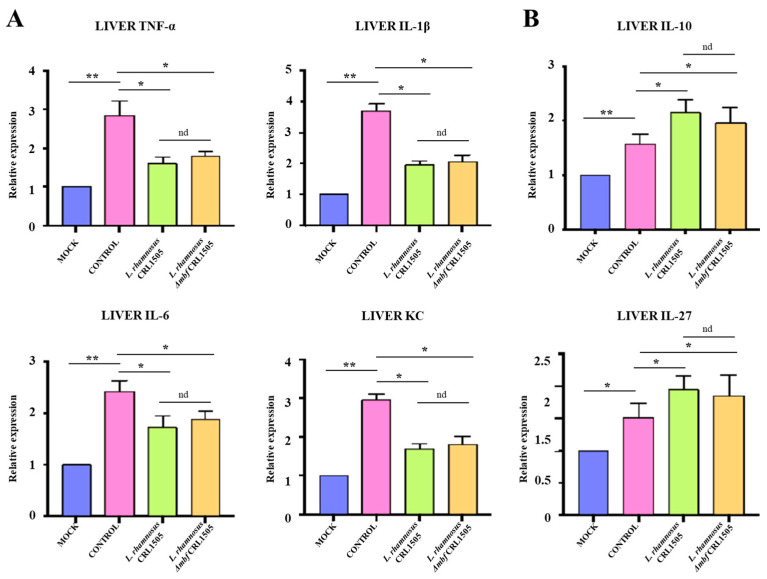
Effect of *Lacticaseibacillus rhamnosus* CRL1505 on liver inflammatory and regulatory cytokines expression after the activation of TLR3. BALB/c mice were orally treated with *L. rhamnosus* CRL1505 or Δ*mbf* strains for five days and then intraperitoneally challenged with poly(I:C). Mice without lactobacilli treatment and stimulated with poly(I:C) were used as controls. Forty eight hours after TLR3 activation, mRNA expressions of (**A**) proinflammatory cytokines *Tnf*, *Il6*, *Cxcl1*, and *Il1b*, and (**B**) regulatory cytokines *Il10*, and *Il27* in liver were determined. Data was presented as means ± SD of experimental groups and analyzed by ANOVA. Significant differences are shown compared to the control group at *p* < 0.05 (*), *p* < 0.01 (**), nd: no significant differences were detected.

We performed liver cytokine analysis to characterize the liver inflammatory response. The liver proinflammatory cytokines *Tnf*, *Il6*, *Cxcl1*, and *Il1b* in control mice were markedly increased after poly(I:C) challenge compared with the mock group (Figure 2A). The increased expression of proinflammatory cytokines was attenuated by both *L. rhamnosus* CRL1505 and *L. rhamnosus* Δ*mbf* CRL1505 pretreatments. In wild-type lactobacilli-treated mice, *Tnf*, *Il6*, *Cxcl1*, and *Il1b expression levels* were 1.8, 1.9, 1.4, and 1.7 fold lower, respectively, compared with poly(I:C)-treated control mice (Figure 2A). Similarly, in mice treated with the mutant strain, *Tnf*, *Il6*, *Cxcl1*, and *Il1b* expression levels were 1.6-, 1.8-, 1.3-, and 1.6-fold lower, respectively, compared with poly(I:C)-treated control mice (Figure 2A).

The expressions of the anti-inflammatory cytokines *Il10*, and *Il27* were also analyzed in liver samples (Figure 2B). Poly(I:C) administration significantly increased *Il10* and *Il27* mRNA expression compared to mock mice. Of note, animals treated with *L. rhamnosus* CRL1505 or *L. rhamnosus* Δ*mbf* CRL1505 exhibited higher levels of both regulatory cytokines than poly(I:C)-treated control mice. In mice treated with the wild-type strain, *Il10* and *Il27* expression levels were 1.7- and 1.8-fold higher, respectively, compared with poly(I:C)-treated controls, whereas both cytokines were approximately 1.8-fold higher in mice treated with the mutant strain (Figure 2B). No significant differences in cytokine expression profiles were observed between the wild-type and mutant CRL1505 strains.

### 3.3. Lacticaseibacillus rhamnosus CRL1505 Administration Differentially Modulate Systemic Cytokines Induced by Poly(I:C)

**Figure 3:**
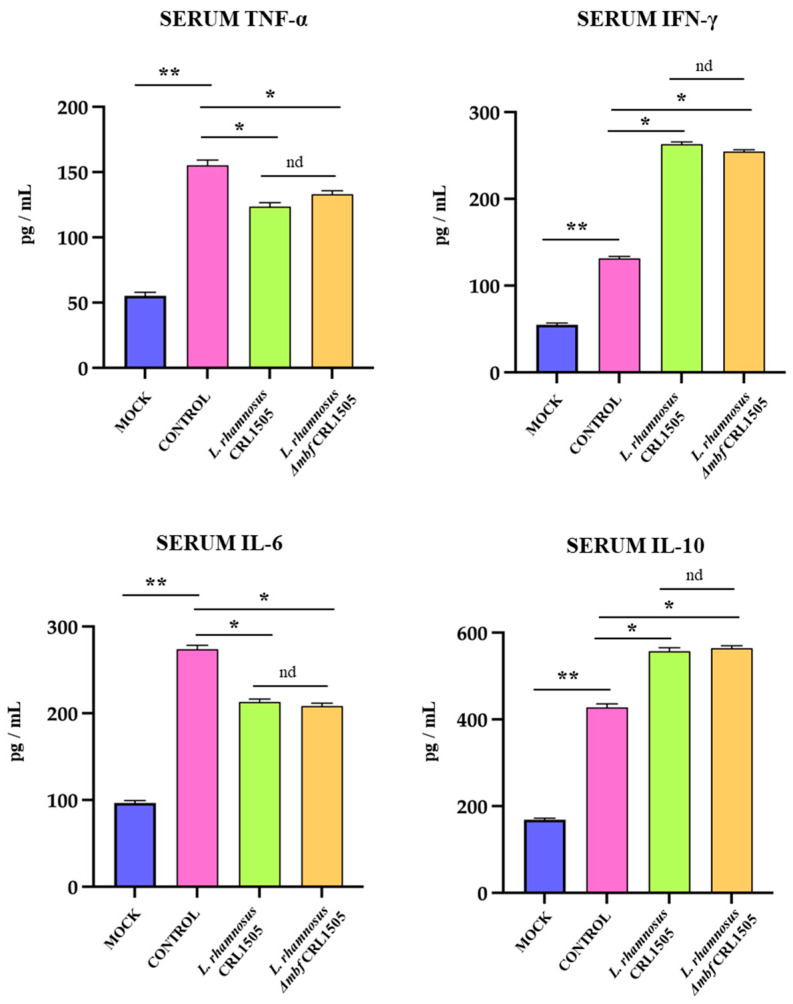
Effect of *Lacticaseibacillus rhamnosus* CRL1505 on serum inflammatory and regulatory cytokines levels after the activation of TLR3. BALB/c mice were orally treated with *L. rhamnosus* CRL1505 or Δ*mbf* strains for five days and then intraperitoneally challenged with poly(I:C). Mice without lactobacilli treatment and stimulated with poly(I:C) were used as controls. Forty eight hours after TLR3 activation, serum levels of proinflammatory cytokines TNF-α, IL-6, IFN-γ, and the regulatory cytokine IL-10 were determined by ELISA. Data was presented as means ± SD of experimental groups and analyzed by ANOVA. Significant differences are shown compared to the control group at *p* < 0.05 (*), *p* < 0.01 (**), nd: no significant differences were detected.

In order to study whether hepatic inflammation had repercussions systemically, pro- and anti-inflammatory cytokines were analyzed in serum samples of the distinct experimental groups (Figure 3). The serum proinflammatory cytokines TNF-α, IL-6, and IFN-γ were markedly increased after poly(I:C) challenge compared with the control group. This effect was attenuated by both *L. rhamnosus* CRL1505 and *L. rhamnosus* Δ*mbf* CRL1505 pretreatments (Figure 3). Additionally, IL-10 was increased in the serum of mice pretreated with wild-type or mutant CRL1505 strains compared to controls (Figure 3). There was no significant difference in the cytokines profile between *L. rhamnosus* CRL1505 and *L. rhamnosus* Δ*mbf* CRL1505 groups.

### 3.4. Lacticaseibacillus rhamnosus CRL1505 Administration Modulate Liver Antiviral Factors Profile Induced by Poly(I:C)

**Figure 4:**
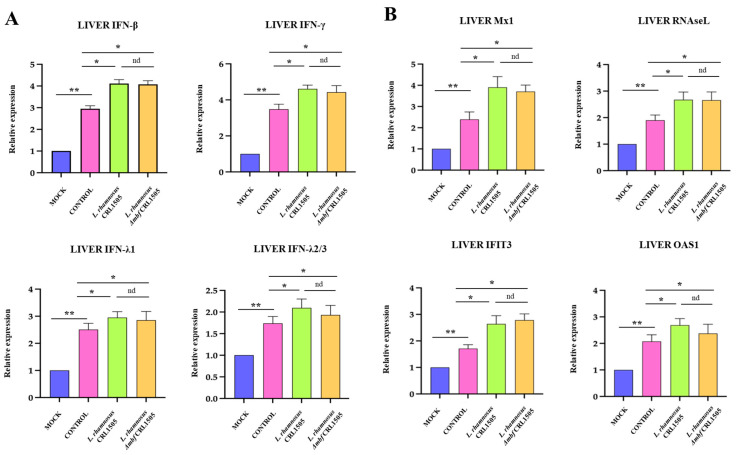
Effect of *Lacticaseibacillus rhamnosus* CRL1505 on liver antiviral factors expression after the activation of TLR3. BALB/c mice were orally treated with *L. rhamnosus* CRL1505 or Δ*mbf* strains for five days and then intraperitoneally challenged with poly(I:C). Mice without lactobacilli treatment and stimulated with poly(I:C) were used as controls. Forty eight hours after TLR3 activation, mRNA expressions of (**A**) interferons *Ifng*, *Ifnb1*, *Ifnl2/3*, and *Ifnl1* and (**B**) antivirals factors *Mx1*, *Oas1*, *Rnasel*, and *Ifitm3* in liver were determined. Data was presented as means ± SD of experimental groups and analyzed by ANOVA. Significant differences are shown compared to the control group at *p* < 0.05 (*), *p* < 0.01 (**), nd: no significant differences were detected.

Finally, since poly(I:C) triggers TLR3 pathway mimicking the reaction generated by viruses, the hepatic mRNA expressions of *Ifng*, *Ifnb1*, *Ifnl2/3*, Ifnl1 (Figure 4A) and the antiviral factors *Mx1*, *Oas1*, *Rnasel*, *Ifitm3* (Figure 4B) were studied in the liver of all the experimental groups. The four IFNs evaluated were increased in the liver tissues after poly(I:C) administration. In agreement, the mRNA expression level of antiviral factors regulated by IFNs were augmented in the liver of poly(I:C)-challenged mice compared to mock animals. Of note, lactobacilli-treated mice showed higher mRNA expression values of the eight antiviral factors when compared to the control group. No significant differences were found between *L. rhamnosus* CRL1505 and *L. rhamnosus* Δ*mbf* CRL1505 groups (Figure 4).

## 4. Discussion

The liver possesses a variety of unique and highly specialized mechanisms that enable it to tolerate external antigens and pathogens, thereby maintaining both innate and adaptive immune balance as well as general hepatic homeostasis [47]. This immune homeostasis is supported by a rich population of resident immune cells [48]. When immune activation becomes excessive, however, it may lead to the development of acute liver injury and associated pathological alterations [49]. Death of hepatocytes through apoptosis, necrosis or necroptosis varies depending on the hepatotropic insult and correlates with both the degree of tissue damage and clinical outcomes [49,50].

Synthetic dsRNA analogs such as poly(I:C) are widely used to model viral RNA sensing in the liver, as they accurately recapitulate the molecular patterns generated during viral replication through the activation of TLR3 signaling [5,7]. In hepatocytes and hepatic immune cells, poly(I:C) engagement of endosomal TLR3 initiates TRIF-dependent signaling cascades that lead to the activation of IRF3 and NF-κB and the subsequent induction of type I IFNs and proinflammatory mediators. Consistent with this mechanism, poly(I:C) administration in control mice increased IL-6, IL-1β, and TNF-α levels, reflecting the well-established TLR3-driven hepatic inflammatory response to dsRNA sensing rather than nonspecific toxicity [8,9]. In addition, increments in the expression of regulatory cytokines in the liver tissue were detected after poly(I:C) administration. This increased *Il10* and *Il27* expression is consistent with the well-established capacity of TLR3 signaling to induce counter-regulatory pathways that limit excessive inflammation. Both cytokines are known to participate in feedback mechanisms that restrain tissue damage during acute antiviral responses [51,52,53]. Importantly, hepatocytes themselves mount robust TLR3-dependent responses to poly(I:C), contributing directly to hepatic inflammation through coordinated signaling involving TRIF, protein kinase R (PKR), and inducible nitric oxide synthase (iNOS), which collectively amplify interferon production, the expression of antiviral genes and inflammatory signaling within the liver microenvironment [5,8]. In line with these findings, our study showed that poly(I:C) administration induced hepatic expression of interferons (IFN-β, IFN-γ, IFN-λ1, IFN-λ2/3) and antiviral factors (Mx1, OAS1, RNaseL and IFITM3). These increments are consistent with the antiviral program triggered by TLR3 recognition of double-stranded RNA, which induces type I and type III IFNs and the subsequent expression of IFN-stimulated genes that establish an antiviral state in hepatocytes and immune cells [10,54,55]. Then, the previous studies and the results presented in this work show that the intraperitoneal administration of poly(I:C) to mice can trigger hepatic inflammation through activation of dsRNA-sensing pathways and therefore serves as a suitable model to study antiviral inflammatory responses in the liver. Of note, although poly(I:C) is widely used as a synthetic analog of viral dsRNA and a classical agonist of TLR3, it is important to acknowledge that in vivo responses to poly(I:C) may involve additional cytosolic dsRNA sensors, including MDA5, RIG-I, and PKR. In the present study, we did not directly assess the specific contribution of TLR3 or TRIF signaling using receptor-deficient models or pathway-specific inhibition. Therefore, our findings should be interpreted in the context of modulation of poly(I:C)-triggered dsRNA-sensing responses rather than exclusive TLR3 dependency. Future studies using genetic or pharmacological approaches will be required to further dissect the relative contribution of individual dsRNA sensors to the observed poly(I:C)-dependent hepatic inflammation.

Immunobiotics represent an emerging strategy for controlling inflammation and enhancing resistance to diseases. Consistent with this concept, probiotics have been widely reported to exert hepatoprotective effects across diverse experimental models of liver disease, largely through modulation of the gut–liver axis. In the context of chronic immune-mediated disorders, probiotic interventions attenuate autoimmune hepatitis in murine models induced by S100/CFA, as demonstrated by reduced inflammatory cell infiltration, decreased serum transaminase activity, and restoration of immune homeostasis through an increase in regulatory T cells accompanied by a reduction in Th1 and Th17 responses [31]. Similarly, in models of metabolic liver disease, immunobiotics can ameliorate metabolic dysfunction-associated steatotic liver disease and liver fibrosis in mice fed a methionine- and choline-deficient diet [56]. These effects include reduced hepatic steatosis and inflammation, lower circulating endotoxin levels and decreased expression of profibrotic mediators such as transforming growth factor-β and α-smooth muscle actin, and are associated with improved intestinal barrier integrity and reduced translocation of lipopolysaccharide from the gut to the liver, leading to suppression of TLR4/NF-κB-dependent inflammatory signaling [56]. In addition, specific probiotic strains such as *Lactobacillus acidophilus* LA14 have been shown to mitigate acute liver injury induced by D-galactosamine or acetaminophen, as reflected by reduced serum AST and ALT levels and attenuated hepatic inflammation. Clinical and preclinical evidence also indicates that probiotics improve intestinal barrier function in patients with compensated cirrhosis and confer protection against liver injury in murine models of sepsis [57].

Growing evidence indicates that probiotics can modulate antiviral immunity in the context of viral hepatitis. In murine models and pilot clinical studies of chronic hepatitis B (CHB), probiotic supplementation has been associated with reduced hepatitis B surface antigen levels and viral replication, accompanied by enhanced intrahepatic CD4^+^ IFN-γ–producing T cell responses and restoration of intestinal homeostasis [58,59,60]. Notably, probiotic-derived metabolites such as spermidine can translocate from the intestine to the liver and reproduce antiviral effects through the induction of autophagy in CD4^+^ T cells, a process tightly linked to innate IFN signaling [60]. These immunomodulatory effects are highly relevant to TLR3-dependent pathways, which play a central role in dsRNA sensing in hepatocytes and hepatic immune cells [61,62,63,64]. Among the immunobiotic strains with antiviral capabilities, *L. rhamnosus* CRL1505 is of particular relevance, as it is used as an immunobiotic specifically formulated for children [65] and has been extensively characterized by our laboratory. Previous studies have demonstrated its capacity to regulate cytokine signaling, enhance the activity of innate and adaptive immune cells, and promote balanced inflammatory responses across different mucosal and systemic contexts [46,66]. Although most investigations have focused on its effects in the intestinal and respiratory immune systems, the breadth of its immunomodulatory activity suggested potential roles beyond these sites. In the present work, we expand this knowledge by evaluating, for the first time, its capacity to modulate inflammation in a poly(I:C)-induced acute hepatitis model. Our findings demonstrate that immunobiotic administration significantly attenuated poly(I:C)-induced liver injury, as evidenced by reduced serum AST and ALT levels and decreased inflammatory cell infiltration, indicating effective modulation of poly(I:C)-induced hepatic inflammation. This was further supported by a marked reduction in systemic proinflammatory mediators, including TNF-α and IL-6, concomitant with increased circulating IL-10 and IFN-γ, suggesting a shift toward a controlled antiviral immune profile. At the hepatic level, the immunobiotic treatment selectively dampened the poly(I:C)-induced upregulation of TNF-α, IL-6, IL-1β, and the chemokine KC, while restoring the expression of regulatory cytokines such as IL-10 and IL-27, which have been shown to limit excessive inflammation by promoting STAT3-mediated anti-inflammatory responses and suppressing proinflammatory T helper differentiation, thereby contributing to immune resolution following innate TLR stimulation [67,68]. Collectively, these results indicate that immunobiotic *L. rhamnosus* CRL1505 does not simply suppress inflammation but rather modulates antiviral and inflammatory responses triggered by dsRNA sensing. In line with this statement, we observed that CRL1505-treated mice have improved hepatic expressions of IFN-γ, IFN-β, IFN-λ1, IFN-λ2/3, Mx1, OAS1, RNaseL, and IFITM3, which have been shown to be of great importance for the resistance against hepatotropic viruses. These findings extend previous observations on the immunomodulatory properties of CRL1505 by demonstrating, for the first time, its capacity to reinforce intrahepatic antiviral gene programs that are critically involved in resistance to hepatotropic viral infections. For example, mice lacking the hepatic type-I IFN receptor (Ifnar1ΔHep) are highly susceptible to HAV infection, displaying viral shedding, elevated transaminases, and hepatic immune infiltration [69]. In addition, it has been shown that functional polymorphisms in the IFN-γ gene, which are associated with low and high producer phenotypes, significantly modify the outcomes of chronic HBV infection [70]. Similarly, studies have linked genetic variants in the IFN-λ chromosomal region [71], polymorphisms of the human MxA, MxB [72], OAS1 and OAS2 [73] genes to a differential resistance to HCV infection and the severity of liver disease. These results encourage future study of the effect of *L. rhamnosus* CRL1505 in models of infections with hepatotropic viruses to determine its ability to limit viral replication through modulation of hepatic antiviral immunity.

It is generally acknowledged that adhesion-related factors play an important role in enabling immunobiotics to elicit beneficial host responses. Previous studies identified the mbf as a key protein potentially involved in mucosal adhesion in the *L. rhamnosus* species. Deletion of mbf in *L. rhamnosus* FSMM22 markedly reduced bacterial adhesion to the extracellular matrix compared with the wild-type strain, supporting its role in intestinal colonization processes [74]. Complementary studies in *L. rhamnosus* GG identified a gene cluster encoding the SpaCBA polymeric pili, which include the mucosal adhesin SpaC at the distal tip. Both, SpaC and Mbf, have been shown to actively participate in adhesion to human mucus [75], highlighting that multiple surface-associated factors contribute to bacterium–epithelium interactions. Consistently, SpaCBA-deficient mutant strains exhibited a marked reduction in adhesion to intestinal epithelial cells and biofilm formation, accompanied by alterations in immune signaling. In vitro studies using Caco-2 cells demonstrated that pili-deficient mutants induced approximately two-fold higher levels of interleukin-8 (IL-8) mRNA compared to the wild-type strain, suggesting that SpaCBA pili exert a modulatory effect that attenuates proinflammatory responses triggered by other bacterial surface components [76].

In this regard, we have previously developed a knockout mutant derived from the CRL1505 strain lacking the *mbf* gene (Δ*mbf* strain), and demonstrated that this mutant bacterium have a reduced capacity to adhere to mucins and intestinal epithelial cells compared to the wild-type strain [43]. Interestingly, studies evaluating intestinal and respiratory antiviral immunity after the oral administration of *L. rhamnosus* Δ*mbf* showed that it had an immunomodulatory effect similar to the observed in the wild-type strain. In this case, the mbf expression is not required for *L. rhamnosus* CRL1505-mediated immunomodulation at the mucosal compartments. Here, we extend these findings by demonstrating that mbf is also not essential for the CRL1505 strain to modulate hepatic antiviral immunity. This observation suggests that modulation of hepatic antiviral responses by CRL1505 can occur independently of bacterial adhesion factors traditionally associated with intestinal colonization.

Despite the strengths of this study, some limitations should be acknowledged. First, the analysis of hepatic antiviral and inflammatory responses was primarily performed at the transcriptional level. Although serum cytokines were quantified at the protein level and supported the immunomodulatory effects observed, the expression of several intrahepatic antiviral mediators was evaluated by qPCR. While transcriptional induction of interferon-stimulated genes is widely accepted as a reliable indicator of antiviral pathway activation in poly(I:C) models, additional protein-level validation of selected hepatic targets would provide further mechanistic insight. Second, we did not directly assess TLR3 expression in liver tissue in the present study. However, the poly(I:C)-induced inflammatory model employed here has been extensively validated as a TLR3-dependent system in previous studies, including genetic approaches using TLR3-deficient animals. Future work incorporating direct assessment of receptor expression and downstream signaling components would contribute to further refine the mechanistic understanding of CRL1505-mediated immunomodulation in this context.

## 5. Conclusions

In the present study, we demonstrate for the first time that the immunobiotic *Lacticaseibacillus rhamnosus* CRL1505 exerts protective effects in a poly(I:C)-induced acute hepatitis model. Preventive administration of the CRL1505 strain to mice significantly reduced poly(I:C)-induced liver injury, as evidenced by decreased serum AST and ALT levels, and was associated with a differential modulation of proinflammatory and regulatory cytokines, as well as interferons and antiviral effector molecules. Notably, the hepatoprotective and immunomodulatory effects of CRL1505 were observed independently of its intestinal colonization capacity, since the Δ*mbf* mutant strain displayed effects comparable to those of the wild-type bacterium. Collectively, these findings highlight the ability of CRL1505 to modulate dsRNA-triggered hepatic immune responses, enhancing antiviral defenses while limiting inflammation-driven liver damage, and support its potential as a complementary dietary-based strategy to mitigate immune-mediated liver injury in the context of viral hepatitis.

## Data Availability

The original contributions presented in this study are included in the article/Supplementary Materials. Further inquiries can be directed to the corresponding authors.

## Supplementary Materials

The following supporting information can be downloaded at: https://www.mdpi.com/article/10.3390/foods15061034/s1, Table S1. Experimental groups and treatment conditions in BALB/c mice.

## Abbreviations

The following abbreviations are used in this manuscript:

ALT	Alanine aminotransferase
AP-1	Activator protein 1
AST	Aspartate aminotransferase
BALB/c	Inbred mouse strain (BALB/c)
CCl4	Carbon tetrachloride
CHB	Chronic hepatitis B
dsRNA	Double-stranded RNA
ELISA	Enzyme-linked immunosorbent assay
HAV	Hepatitis A virus
HBV	Hepatitis B virus
HCV	Hepatitis C virus
HDV	Hepatitis D virus
HEV	Hepatitis E virus
IFITM3	Interferon-induced transmembrane protein 3
IFN	Interferon
IFN-β	Interferon beta
IFN-γ	Interferon gamma
IFN-λ	Type III interferon
IKKε (IKK-i)	IκB kinase epsilon
IL	Interleukin
IP	Intraperitoneal
IRF3/7	Interferon regulatory factor 3/7
KC	Keratinocyte-derived chemokine (CXCL1)
mbf	Mucus-binding factor
MRS	de Man–Rogosa–Sharpe (culture medium)
Mx1	Myxovirus resistance protein 1
NAFLD	Non-alcoholic fatty liver disease
NAP1	NF-κB-activating kinase-associated protein 1
NF-κB	Nuclear factor kappa B
OAS1	2′-5′-Oligoadenylate synthetase 1
PBS	Phosphate-buffered saline
PCR	Polymerase chain reaction
PKR	Protein kinase R
poly(I:C)	Polyinosinic:polycytidylic acid
RIP1	Receptor-interacting protein 1
RNaseL	Ribonuclease L
SD	Standard deviation
STAT3	Signal transducer and activator of transcription 3
TBK1	TANK-binding kinase 1
TLR	Toll-like receptor
TLR3	Toll-like receptor 3
TNF-α	Tumor necrosis factor alpha
TRAF	TNF receptor-associated factor
TRIF	TIR-domain-containing adapter-inducing interferon-β
