# Supplementary material for: Lacticaseibacillus rhamnosus CRL1505 Ameliorates Liver Injury and Inflammation in Poly(I:C)-Induced Acute Hepatitis
Source: Foods. 2026 Mar 16;15(6):1034. doi: 10.3390/foods15061034 (PMC13025889; doi:10.3390/foods15061034)
Supplement: Supplementary file 1 [file foods-15-01034-s001.zip › foods-4175922-supplementary.pdf]

Supplementary Table S1. Experimental groups and treatment conditions in BALB/c mice. Six-week-old BALB/c mice were orally treated for five consecutive days with the indicated immunobiotic strains or vehicle. On day 6, mice were intraperitoneally challenged with the TLR3 agonist poly(I:C) (30 μg in 100 μl PBS) or PBS (mock group). The antiviral innate immune response was evaluated 48 h after stimulation.

Group	*n* (mice)	Oral treatment (5 days)	Vehicle	Poly(I:C) challenge	Route	Final purpose
Mock	6	None	10% sterile non-fat milk	PBS	Intraperitoneal	Baseline control (no TLR3 activation)
Control	6	None	10% sterile non-fat milk	Poly(I:C) 30 μg in 100 μl PBS	Intraperitoneal	Positive inflammatory control
CRL1505	6	*L. rhamnosus* CRL1505 (10^8 cells/mouse/day)	10% sterile non-fat milk	Poly(I:C) 30 μg in 100 μl PBS	Intraperitoneal	Evaluation of immunobiotic preventive effect
Δ*mbf* CRL1505	6	*L. rhamnosus* Δ*mbf* CRL1505 (10^8 cells/mouse/day)	10% sterile non-fat milk	Poly(I:C) 30 μg in 100 μl PBS	Intraperitoneal	Evaluation of the *mbf* mutant strain
